# Editorial: Pre-Conference Research Topic: 16th International Symposium on Schistosomiasis

**DOI:** 10.3389/fimmu.2021.774311

**Published:** 2021-11-25

**Authors:** Cristina Toscano Fonseca, Thiago Almeida Pereira, J. Russell Stothard, Roberta Lima Caldeira, Marina Moraes Mourão

**Affiliations:** ^1^ Grupo de Biologia e Imunologia de Doenças Infecciosas e Parasitárias, Instituto René Rachou, Fundação Oswaldo Cruz, Belo Horizonte, Brazil; ^2^ Institute for Stem Cell Biology and Regenerative Medicine, Stanford University School of Medicine, Stanford, CA, United States; ^3^ Department of Tropical Disease Biology, Liverpool School of Tropical Medicine, Liverpool, United Kingdom; ^4^ Grupo de Helmintologia e Malacologia Médica, Instituto René Rachou, Fundação Oswaldo Cruz, Belo Horizonte, Brazil

**Keywords:** host-parasite interactions, vaccines, immunobiology, Biomphalaria sp., Schistosoma sp, schistosomicides, burden of disease, schistosomiasis control

Traditionally, every two years, the International Symposium on Schistosomiasis, organized by Fundação Oswaldo Cruz, takes place in Brazil. The Symposium brings together scientists from all over the world working on different aspects of schistosomiasis. As this disease affects approximately 240 million people worldwide (1), it is crucial to appraise recent advances in schistosome biology, parasite interactions with hosts, and review progress in the development and evaluation of new tools for disease diagnosis, treatment, and control.

The 16th edition of the Symposium (http://www.vppcb.fiocruz.br/16symposium-schisto/_en) was scheduled to take place in August 2020 but was postponed to November 2022, due to the COVID-19 pandemic. The published papers in this Research Topic sustain our global engagement and aim to keep the research momentum on schistosomiasis, a neglected tropical disease, thriving.

Our infographic highlights our international engagement across 23 countries with a total of 177 authors (**Figure 1**). Of note, whilst intestinal schistosomiasis (*Schistosoma mansoni*) occurs in South America and still poses a significant public health challenge in parts of Brazil, it also occurs in Africa, alongside urogenital schistosomiasis (*Schistosoma haematobium*). In Asia, however, intestinal schistosomiasis is caused by a different schistosome species (*Schistosoma japonicum*), and the appreciation of this is essential to ensure that global research and control efforts are appropriate and complementary.

**Figure 1:**
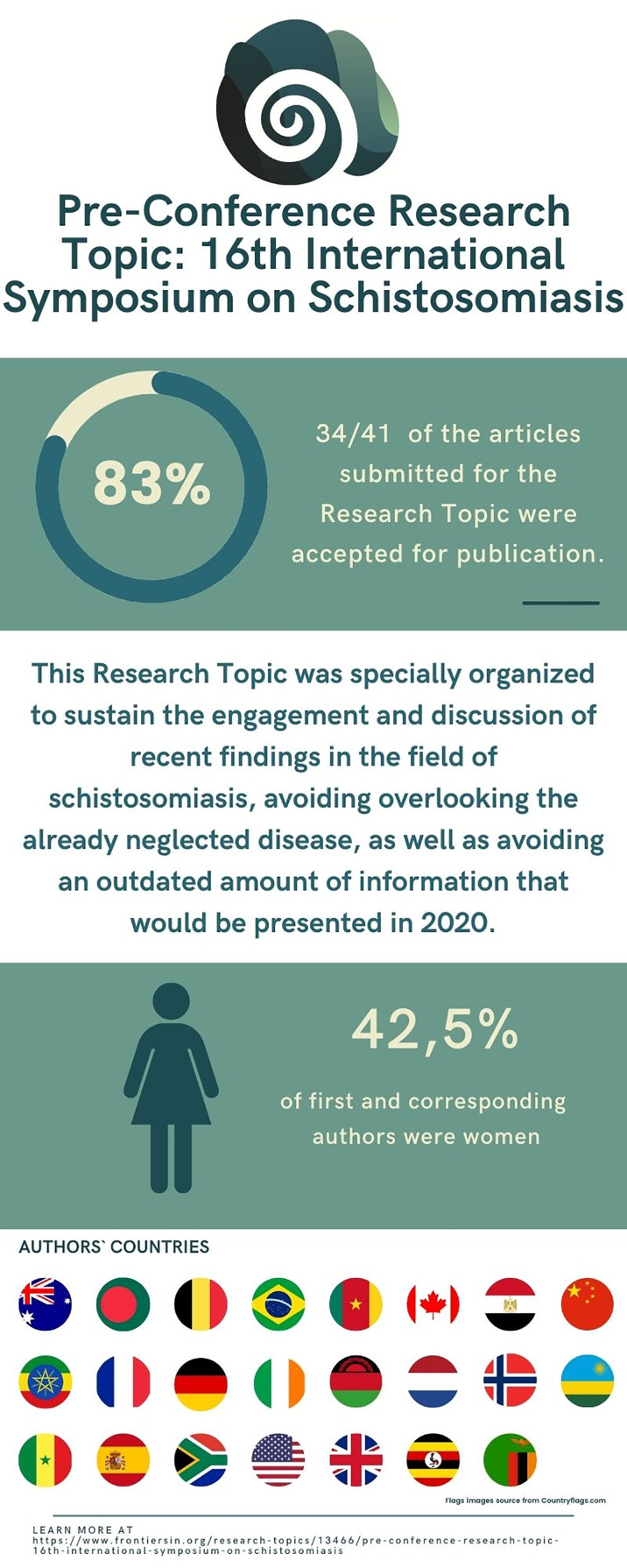
Pre-conference research topic of the 16th International Symposium on Schistosomiasis, in numbers. The infographic summarizes the most relevant information regarding the research topic main goal, articles’ acceptance rate, and authors’ profile.

## Schistosome Biology

*Schistosoma *spp. are the etiological agents of all forms of schistosomiasis, and each species has a complex life cycle involving various species of aquatic mollusk(s) as the intermediate host, with various mammal(s) acting as definitive hosts. Thus, understanding schistosome biology and its interaction with its hosts is crucial for developing tools and strategies for disease control and elimination.

In this context, Hambrook and Hannington have gathered literature, highlighting what they call “the foundational work,” which demonstrates the multitude of challenges that the host poses for the schistosome and the ways that they have evolved to mitigate or overcome these challenges. Next, they explore how the different parasite life stages deal with the diverse immune systems they encounter in invertebrate and vertebrate hosts. Finally, they delineate the invertebrate host immune responses and parasite survival strategies in the definitive host, including antigenic mimicry, variability of surface molecules, production of host hormones and products (E/S P) able to inhibit host response, degradation of complement components, incorporation of host antigens, and prevention of blood clots.

Our understanding of senescence in schistosomes is becoming more complex as the cellular biology of the worm is probed. You et al. bring together recent advances in schistosome stem cells research. They review two classes of stem cells, germinal cells involved in asexual reproduction (intramolluscan) and the neoblasts in sexual reproduction, which occur in intramammalian stages. The authors also discuss the use of these resources as a new tractable platform to underpin more general research on schistosomiasis.

## Adaptation in Parasitism

There are still knowledge gaps today regarding many aspects of schistosome biology and how worms respond to the humoural environment within each intermediate and/or definitive host. Anisuzzaman et al. propose a new methodology to investigate the development and survival of the intravascular stage of *S. mansoni*. In their article, the adult worms were cultivated in the presence of serum from mice and other different mammalian species to interrogate *in vivo *the importance of soluble complement system factors. Despite not revealing a notable role for soluble proteins from the mouse complement system, this article will benefit the scientific community in designing new studies to investigate the immunological mechanisms defining host susceptibility to *S. mansoni* infection.

Turning attention to the mollusk, bioinformatic analyses by Pinaud et al. reveal 23 biomphalysin-like genes in *Biomphalaria glabrata*, the snail host for *S. mansoni*, and the presence of highly similar sequences on the same scaffolds indicate recent duplications. The relative absence of differences in sequence signatures leads the authors to refer to the likely HGT origin of biomphalysins as old domestication. Then the structures of the small lobe (domain I) of different biomphalysins showed similarities to immune-type factors that interact with antigens. Then, pathogen challenge modifies expression patterns, and protein level interactome analyses indicate that categories of pathogens are bound by specific subsets of biomphalysins.

## Advances in Immunobiology

In their review article, Houlder et al. provide a clear and in-depth description of our current knowledge of the biology of the parasite, the definitive host immune response, and the pulmonary pathology during the acute and chronic phase of the disease. The authors also highlight important questions that still need to be answered by future studies. The roles of monocytes and macrophages in the response against schistosomes and in disease pathogenesis are reviewed by Souza et al. and emphasis is given to the alternatively activated macrophages’ function in host protection and pathogenesis.

In an attempt to better understand the immune response of humans with schistosomiasis mansoni and its relationship with disease burden, Santana et al. characterize the CD4+ T cell profile of patients with different degrees of liver fibrosis. Patients with periportal fibrosis had an increase of Th2 cytokines IL-4, IL-5, and IL-13, and higher levels of IL-10 but a lower IL-10/IL-13 ratio than patients without fibrosis. Interestingly, IL-17 was also increased in patients with periportal fibrosis, and TGF-β production correlated with fibrosis, thus reiterating that schistosomiasis fibrosis pathogenesis is complex and induced by several pathways, including the IL-13/IL-17/TGF-β axis. Therefore, multi-target therapy should be preferred over monotherapy to impair fibrogenesis and ameliorate schistosomiasis fibrosis and portal hypertension.

Understanding the immune response dynamics and their relationship with disease pathogenesis in the murine model is extremely important to develop effective antifibrotic therapies. Song et al. provide an interesting kinetic analysis of the immunological aspects of liver fibrosis induced by carbon tetrachloride (CCl_4_) and by *S. japonicum *infection. This direct comparison of murine schistosomiasis fibrosis with an established fibrotic model largely used to test novel antifibrotic therapies is relevant since several drug candidates manipulate the immune response. In the* S. japonicum *model, a shift from Th1 immunity to Th2/Th17/Treg immunity immunity was observed, reinforcing the role of IL-13 and IL-17 in the pathogenesis of schistosomiasis fibrosis. In contrast, CCl_4_ induced fibrosis is characterized by a Th1 immune response. Therefore, for schistosomiasis, dual targeting of IL-13 and IL-17 might be a better strategy to block fibrogenesis and avoid rebound inflammation.

The study of Da’dara et al. demonstrates that the *S. mansoni* ectoenzyme alkaline phosphatase (SmAP) could cleave the active form of vitamin B6 (PLP) into its intermediate, pyridoxal. Additionally, schistosome enzymes that may be involved in intracellular catabolism of vitamin B6 were identified. Therefore, the authors speculate that the metabolism of the host PLP by SmAP can regulate its concentration in the blood, thus regulating the inflammatory process that utilizes this vitamin.

## The Burden of Disease and Disabilities

To this date, only a few studies have evaluated the impact of severe forms of schistosomiasis on the health-related quality of life of affected patients, and none have included schistosomal myeloradiculopathy. This has limited accurate measurements of disease burden in endemic areas and management of patients with severe forms of the disease. Roriz et al. demonstrate that patients with hepatosplenic schistosomiasis have three times higher chances to have lower scores of overall quality of life, while patients with schistosomal myeloradiculopathy have five times more chances to have worse quality of life scores than healthy adults with similar socio-demographic backgrounds. This study reinforces the importance of eradicating this disease and suggests that multidisciplinary clinical management of schistosomiasis patients would be more appropriate to improve patients’ quality of life.

Another complication of chronic schistosomiasis is pulmonary arterial hypertension. In the review published by Sibomana et al., the authors describe our current understanding and knowledge gaps related to pulmonary arterial hypertension caused by schistosomiasis. Their review includes discussions of disease definition and epidemiology, current diagnostic and treatment protocols, prognosis, and insights into disease pathogenesis.

## Co-Infections, Microbiota Interaction, Nutrition Status and Carcinogenesis

Schistosomiasis has been described to impact the outcome of co-infections and nutritional status, and *vice versa* (2, 3). In this regard, Maciel et al. demonstrate that the *S. mansoni* infection in malnourished mice does not impact parasite survival but impacts parasite fecundity and the size and development of granuloma. On the other hand, the authors show that *S. mansoni* infection aggravates mice nutrition status. Miranda et al.‘s findings suggest that a history of American Tegumentary Leishmaniasis (ATL) may increase susceptibility to *S. mansoni* infection, as a higher prevalence of intestinal schistosomiasis was observed in individuals with a previous history of ATL. Individuals with active intestinal schistosomiasis with a previous history of ATL presented a modulated immune response that may increase susceptibility to schistosomiasis but may also be beneficial to reduce disease morbidity.

Continuing the discussion on host-parasite interactions, Cortés et al. describe the putative association of the gut microbiota with *S. mansoni* infection. They equate the influence of the parasite on the microbiota composition in mice, humanized and indigenous. The work demonstrates that these two models react differently to infection and suggests that taxa abundance and diversity of the microbiota could influence the development of *S. mansoni* infections.

All forms of schistosomiasis are associated with the development of certain types of cancer, but the mechanisms involved are not fully understood, particularly with regard to *S. japonicum* infection and colorectal cancer pathogenesis. Wu et al. elegantly demonstrate that SjE16.7 protein from the eggs of *S. japonicum* binds to RAGE (Receptors for Advanced Glycation End Products) in host cells inducing NF-kB activation, increases in oxidative stress and inflammation with high levels of IL-6 and TNF, and promotes colorectal cancer in mice. This relevant study provides novel information regarding the host-parasite interactions that contribute to the pathogenesis of inflammation and carcinogenesis mediated by helminths.

## Genital Schistosomiasis

In urogenital schistosomiasis, it is hypothesized that the cervicovaginal inflammation caused by *S. haematobium* eggs may contribute to HIV-1 infection, leaving women from those endemic areas more susceptible. However, the mechanisms behind the possible association between Female Genital Schistosomiasis (FGS) and HIV-1 infection are largely unknown. Sturt et al. demonstrate that women with higher FGS burden also have an increase in Th2 cytokines and the pro-inflammatory cytokine IL-15 compared to the control group without FGS, thus confirming that FGS alters the immune environment of the cervicovaginal mucosa and may contribute to HIV-1 infection. Thus, this pioneering study sheds some new light on the mechanisms involved in the association of FGS and HIV-1 infection observed in sub-Saharan women.

Across genders, Male Genital Schistosomiasis (MGS) is an understudied manifestation of *S. haematobium* infection and, similar to FGS, is associated with HIV infection. However, few epidemiological studies are available, even in highly endemic areas in sub-Saharan Africa. Kayuni et al. conduct a large longitudinal cohort study to investigate the prevalence of MGS among fishermen from Lake Malawi. This work provides valuable information for public health programs for schistosomiasis control and calls attention to the need to investigate this condition in *S. haematobium* endemic areas and facilitate access to treatment in combination with education and awareness programs targeted to men in order to provide early detection of MGS and avoid progression of this condition.

## A Focus on New Diagnostics

Many efforts have recently been devoted to developing, validating, and evaluating schistosomiasis diagnosis methods, mainly because it has become essential for disease surveillance and monitoring the effectiveness of elimination actions. In this context, Siqueira et al. evaluate the efficacy of a Real-time PCR assay to detect *S. mansoni* infection in individuals with low parasite burdens from two different endemic areas and investigate if this assay could also be used as a post-treatment cure control method. Compared with the reference test (24 slides Kato-Katz + Saline Gradient analysis), the real-time PCR assay had satisfactory performance and could be a powerful tool to improve diagnosis and cure assessment in low burden endemic infection areas.

Cavalcanti et al. also applied different diagnostic tests in seven cases of acute schistosomiasis in children living in a non-endemic area. The clinical manifestation and laboratory analysis reports before and after Praziquantel treatment bring insights to the accuracy of different tests in acute schistosomiasis diagnosis and assessments of cure after treatment. The results also demonstrate a treatment regimen that resulted in a parasite-free status in these children.

## Novel Treatments and Preventions Against Schistosomiasis

Schistosomiasis treatment and the discovery of new schistosomicide drugs are still areas of great interest in the field. Although Praziquantel is a safe and effective anti-schistosomal drug, it is ineffective against juvenile worms, and there is growing evidence of Praziquantel resistance in some endemic areas. Therefore, novel efficient therapies are needed. Moreira-Filho et al.‘s state-of-the-art review on the current tools and novel strategies that have evolved in the past few years will be an important resource for the field of drug discovery for schistosomiasis. In addition, the detection of low burden individuals and accurate cure assessment is still a challenge in schistosomiasis, particularly in areas where Mass Drug Administration (MDA) control programs are in place.

In the mini-review published by Tallima et al., the authors have gathered evidence that points to Arachidonic Acid (ARA) as a schistosomicide that can be used to treat *S. mansoni* and *S. haematobium* infection. Additionally, the authors describe the critical role of ARA in the protective mechanisms induced by vaccines using cysteine-peptidase in their formulation, since ARA activates the parasite tegument-associated neutral sphingomyelinase, which promotes the cleavage of tegument sphingomyelin, thus allowing antigen exposure and antibody-dependent cell-mediated cytotoxicity.

Mu et al. provide an up-to-date review on the use of schistosome-derived products as potent immunomodulators that could be used therapeutically to treat or ameliorate the sequelae of chronic inflammatory diseases such as allergic asthma, arthritis, colitis, diabetes, sepsis, cystitis, and cancer. This pertinent, concise paper highlights the flourishing research field of immunomodulation and presents the most compelling evidence for the promising beneficial aspects of using parasite-derived molecules in treating chronic inflammation.

In an attempt to accelerate bench-to-bedside delivery of a vaccine against schistosomes, Sanches et al. demonstrate that an *in silico* approach could be an effective and potent strategy to design a multi-epitope chimeric vaccine against *S. mansoni* infection. Using a robust immunoinformatics approach and prediction models, the authors provide a novel vaccine candidate that could provide a robust humoral and cellular response and therefore provide an important tool to eliminate schistosomiasis. *In vivo* studies would be required to test novel candidates and confirm if this novel strategy would accelerate the translation to the clinic.

Concerning pre-clinical studies of vaccines, Perera et al.’s work demonstrates that *S. mansoni* Cathepsin, a parasite gut peptidase, when formulated with two new adjuvants (sulfated lactosyl archaeol (SLA) archaosomes or AddaVaxTM), was able to induce a protective response that significantly reduced parasite burden, granuloma size, and the amount of miracidium hatching from the eggs that were eliminated within the feces of immunized mice. The data obtained in this pre-clinical trial suggest that these vaccine formulations might be helpful in controlling schistosomiasis morbidity and transmission. However, clinical trials are necessary to evaluate its safety and efficacy in humans.

## Schistosomiasis Control and Public Health

Schistosomiasis control and elimination is still a challenge, despite intense research and investment. Our collection received several articles that cover this topic. Wang et al. retrospectively analyze clinical and epidemiological data of imported schistosomiasis in China from 1979 to 2019. They found that 78 and 262 cases of schistosomiasis caused by* S. mansoni *and *S. haematobium* infection, respectively, were reported in Chinese and African citizens coming from Africa. Additionally, 15 cases of schistosomiasis caused by an unknown species are also reported. The authors describe in their study the most common clinical manifestations of the disease and the diagnosis methods employed in infection identification. The study raises two concerns, the delay in the correct diagnosis of infections by Chinese clinicians that are not alert to the occurrence of disease cases and the presence of the intermediate host for *S. mansoni* in some Chinese provinces that can lead to autochthonous infection cases and the spread of the disease in these regions.

Onasanya et al. discuss the current strategies for schistosomiasis control in sub-Saharan African regions and the main constraints based on these strategies to achieve the expected goals of the WHO 2030 Roadmap, which aims at yearly reductions in morbidity, prevalence, transmission, towards schistosomiasis elimination by 2030. Most importantly, the authors draw attention to the need to design new integrative approaches, considering the disease’s multifactorial spectrum and context particularities in endemic settings. In addition, these strategies should focus on locally appropriate and acceptable ways to increase awareness, and reduce transmission and infection, and equitable ways of treating the disease.

The case for schistosomiasis japonica elimination seems to be even more complex due to its zoonotic characteristics with 46 different animal reservoirs. Li et al., in their concise review, present a historical perspective of the control program in the Dongting Lake Region of China. The authors highlight the strategies used, the impact of the Three Gorges Dam, new insights into the transmission and complexity of the animal reservoirs in the epidemiology of the disease, novel strategies for control, challenges, and areas that need further development in order to achieve elimination. This paper provides essential lessons that could be used in other public health programs in endemic areas to improve schistosomiasis control and eventually eliminate this disease.

Mawa et al. provide a much-needed and timely discussion of schistosomiasis morbidity and the puzzling data on biological hotspots, areas that remain with high transmission and morbidity rates despite control efforts. This critical review discusses the host-parasite-environmental factors that may explain the biological hotspots that prevent successful control of disease burden and is a great challenge for elimination. Only with a proper understanding of the dynamics of host-parasite interactions and particularities of each endemic area will we be able to create and tailor the best strategies that culminate with the elimination of schistosomiasis in such hotspots.

The geographical distribution of *S. mansoni* is tightly tied to the presence of susceptible species of *Biomphalaria* freshwater snails that support the parasite’s transformation into infective stages. Furthermore*, Biomphalaria *spp.* *has shown strong local and global dispersal capacities that may increase due to the global warming phenomenon and increased land development. In this regard, Habib et al. has provided a comprehensive review on *Biomphalaria *spp., their diversity, geographical distribution, ecological and environmental determinants, dispersal and invasion potential, the risk for schistosomiasis transmission, and implications for surveillance. Also, the authors call for effective control initiatives for schistosomiasis, including periodic malacological surveys for the detection of *Biomphalaria *in new potential habitats and identification of species and infection status of collected snails.

Novel approaches in malacological surveying and taxonomy are also present in our collection. Valladares et al. evaluate the power of near-infra-red spectroscopy (NIRS) to assess the chemical phenotype of *B. glabrata*, *B. tenagophila*, and *B. straminea* that are intermediate hosts for schistosomes in Brazil. This study is a proof-of-concept of an innovative, cost-effective, fast, and non-destructive method to discriminate quite similar snails in the field.

Tallam et al. investigate the use of machine learning algorithms – convolutional neural networks (CNN) – in public health using schistosomiasis as a model and showed the usefulness of CNN for classifying parasites and their intermediate host snails. Snails were classified on four neurons and parasite classification *via* eleven neurons. The model was trained with 80% of the data set, and the validation on the remaining unseen 20%. An impressive 99.6% accuracy was obtained for the four snail genera and 91.21% accuracy for the 11 parasite morphospecies. Thus, the CNN achieved superior performance to a trained parasitologist, although the authors suggest that this might be an unfair comparison.

In line with spatial epidemiological themes, the mapping of geospatial risk for the presence of intermediate hosts, Coelho et al. describe an integrated approach to map the environmental conditions. They suggest the use of coliform bacteria as a proxy for fecal contamination. This methodological approach could identify high-risk areas, particularly in lower prevalence settings, which may be important for future control efforts, although some additional considerations are needed for *S. haematobium*, which is passed in the urine.

## Future Expectations

This pre-conference Research Topic of 34 articles published by Frontiers in Immunology, Medicine, Microbiology, and Public Health is a valuable initiative to communicate information to the schistosomiasis research community. This is even more important during these atypical pandemic times to propel the needed advances towards sensitive diagnoses, control, and prevention of this significant disease in neglected populations worldwide. On behalf of the organizing committee, we invite all the schistosomiasis researchers to attend the 16th edition of the International Symposium on Schistosomiasis (November 21-23, 2022) to engage in further discussions.

## Author Contributions

CF, TP, MM, and RC wrote and revised the Editorial. JS revised the Editorial. CF made the infographic (**Figure 1**). TP, MM, RC, and JS revised the infographic. All authors contributed to the article and approved the submitted version.

## Funding

Productivity Fellowship from Conselho Nacional de Desenvolvimento Científico e Tecnológico–Brasil (CNPq) (Grant 303131/2018-7 granted to CTF and 302518/2018-5 granted to MMM).

## Conflict of Interest

The authors declare that the research was conducted in the absence of any commercial or financial relationships that could be construed as a potential conflict of interest.

## Publisher’s Note

All claims expressed in this article are solely those of the authors and do not necessarily represent those of their affiliated organizations, or those of the publisher, the editors and the reviewers. Any product that may be evaluated in this article, or claim that may be made by its manufacturer, is not guaranteed or endorsed by the publisher.
